# Probiotic legacy effects on gut microbial assembly in tilapia larvae

**DOI:** 10.1038/srep33965

**Published:** 2016-09-27

**Authors:** Christos Giatsis, Detmer Sipkema, Javier Ramiro-Garcia, Gianina M. Bacanu, Jason Abernathy, Johan Verreth, Hauke Smidt, Marc Verdegem

**Affiliations:** 1 Aquaculture and Fisheries Group, Wageningen University, De Elst 1, 6708 WD Wageningen, The Netherlands; 2 Laboratory of Microbiology, Wageningen University, Stippeneng 4, 6708 WE Wageningen, The Netherlands; 3 Laboratory of System and Synthetic Biology, Stippeneng 4, Wageningen 6708 WE, The Netherlands; 4 TI Food and Nutrition (TIFN) P.O. Box 557, 6700 AN, Wageningen 6703 HB, The Netherlands; 5 USDA-ARS, Hagerman Fish Culture Experiment Station, 3059F National Fish Hatchery Road, Hagerman, Idaho 83332, USA

## Abstract

The exposure of fish to environmental free-living microbes and its effect on early colonization in the gut have been studied in recent years. However, little is known regarding how the host and environment interact to shape gut communities during early life. Here, we tested whether the early microbial exposure of tilapia larvae affects the gut microbiota at later life stages. The experimental period was divided into three stages: axenic, probiotic and active suspension. Axenic tilapia larvae were reared either under conventional conditions (active suspension systems) or exposed to a single strain probiotic (*Bacillus subtilis*) added to the water. Microbial characterization by Illumina HiSeq sequencing of 16S rRNA gene amplicons showed the presence of *B. subtilis* in the gut during the seven days of probiotic application. Although *B. subtilis* was no longer detected in the guts of fish exposed to the probiotic after day 7, gut microbiota of the exposed tilapia larvae remained significantly different from that of the control treatment. Compared with the control, fish gut microbiota under probiotic treatment was less affected by spatial differences resulting from tank replication, suggesting that the early probiotic contact contributed to the subsequent observation of low inter-individual variation.

The gut microbiota influences a wide range of biological processes in humans^1^,^2^, domesticated terrestrial animals^3^,^4^ and fish^5^,^6^. In fish, despite the significant contribution of several studies on gut microbiota, the current understanding of the functional significance of microbial fluctuations lags well behind that of terrestrial vertebrates. Apart from a few pathogens, host-microbe interactions in fish remain poorly understood. One reason is that the fish gut microbiota is dependent on the aquatic environment. Furthermore, compared with terrestrial animals that undergo embryonic development within an amnion, fish larvae are released into the water at an early ontogenetic stage, when their digestive tract is not yet fully developed and their immune system incomplete^7^. Thus, the use of probiotics in aquaculture is particularly effective during early ontogenetic stages, where large mortalities are commonly observed.

Live microorganisms that confer a health benefit to the host have been demonstrated as useful in aquaculture^8^. Probiotics reduce infections caused by bacterial pathogens^7^,^9^ and have been successfully used as immunostimulants^10^,^11^,^12^ and growth promoters in fish and shrimp^8^,^13^. However, probiotic strains often only transiently colonize the gut and quickly fall below detection limits^7^,^14^,^15^,^16^,^17^,^18^. For ingested bacteria to proliferate and persist within “resident” microbiota, these microorganisms must adapt to the environmental conditions inside the gut, such as nutrient availability, pH and digestive enzymes^19^. The ability of a probiotic strain to survive and successively proliferate in the gut after the cessation of probiotics administration is both host- and probiotic strain-dependent and is highly determined by the mode and duration of administration (through water or feed)^8^. Based on the present level of understanding, the colonization dynamics of fish gut microbiota remain largely stochastic and are affected by gut habitat (i.e., physiology, anatomy) and host genotype^20^,^21^,^22^. Yin *et al*.^23^ suggested that in newly hatched chicks, it is possible to steer gut microbiota by feeding bacterial diets (caecal inocula), leading to the development of distinct communities. In a recent study, we observed that tilapia larvae fed with different microbial diets (sludge-based) developed distinct gut microbiota, although all larvae also shared a large number of species^24^. It is likely that those shared species resulted from larval contact with a common water source after hatching and prior to the first feeding. However, the host-specificity for a particular microbial species modulated by selective pressures within the host gut cannot be excluded.

*Bacillus subtilis* is a Gram- and catalase-positive, rod-shaped, facultative, anaerobic and endospore-forming bacterium observed in air, water, soil and the gastrointestinal tract of humans and animals^25^,^26^,^27^,^28^. Several *Bacillus* spp. (including *B. subtilis*) have been commonly used as probiotics in aquaculture, reflecting their antimicrobial activity against common fish and shrimp pathogens. The beneficial properties of these microbes primarily reflect immune system enhancement (phenoloxidase activity, phagocytic activity and clearance efficiency), competitive exclusion or antibacterial substance production^28^,^29^,^30^,^31^,^32^,^33^,^34^,^35^,^36^,^37^,^38^. In addition, probiotic treatment with *B. subtilis* enhanced the growth and viability of beneficial lactic acid bacteria in the guts of humans and animals^39^.

Prior to investigating the potential probiotic properties of *B. subtilis* in Nile tilapia, the scope of the present study was to assess the impact of the early microbial contact of tilapia larvae on the tilapia gut microbial assembly during later ontogenetic stages. We hypothesized that administration of the probiotic strain to fish larvae early in life, when the gut microbiota is still developing, enhances gut-colonization success and therefore leads to the development of distinct gut communities, even after the fish are exposed to conventional husbandry conditions in active suspension systems.

## Methods

### Ethics statement

This experiment was performed in accordance with Dutch regulations regarding the use of experimental animals and approved by the Ethical Committee of Wageningen University for animal experiments (Project Name: Promicrobe; Registration code: 2011076.c).

### Experimental design

**Figure 1:**
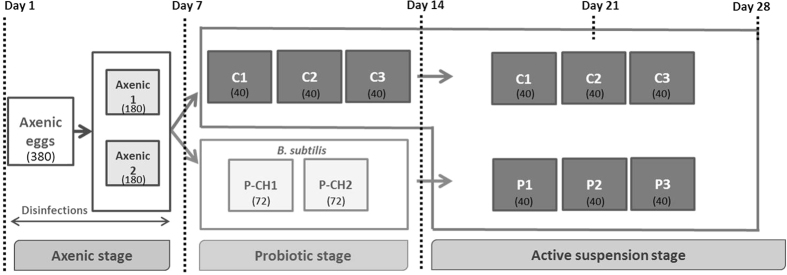
Experimental set-up during the 28-day experimental period. The period was divided into three different stages: Axenic, Probiotic and Active suspension. The numbers in parentheses indicate the initial number of eggs/larvae distributed in the tanks/chambers at each experimental stage. P-CH: Probiotic chamber, C: control treatment, P: Probiotic treatment. 1, 2 and 3: Replicate tanks 1, 2 and 3.

The experimental period was divided into three stages: axenic, probiotic and active suspension. The first two stages were conducted under laboratory conditions, while the third stage was conducted under normal rearing conditions at the Aquatic Research Facility of CARUS, the Animal Experimental Facility of Wageningen University. The total experimental period was 28 days, which is considered sufficient for major ontogenetic changes to occur in tilapia until larvae enter the early juvenile stage^40^. First, from two days post-fertilization, the eggs were reared under axenic conditions for seven days (Days 1–7). Subsequently, the axenic larvae were split into two groups. Half of the larvae was divided over three replicate active suspension tanks, i.e., C1–3 (from day 8 to 28), while the other half was divided over two probiotic chambers (P-CH1 and P-CH2). Probiotic bacteria were supplied for seven consecutive days (days 8–14), after which the larvae from these probiotic chambers were divided over three active suspension tanks (P1-3), where these fish were further raised for another 14 days (day 15–28) (Fig. 1). Throughout the text, “Control (C) treatment” refers to the axenic larvae directly transferred into xenic active suspension tanks, whereas “Probiotic (P) treatment” refers to the axenic larvae initially exposed to the probiotic strain and subsequently transferred to active suspension tanks.

### Axenic conditions

To diminish the effects of water microbiota on early gut colonization, the larvae were initially reared under axenic conditions according to Situmorang *et al*.^41^. Briefly, two days post-fertilization eggs were washed from the mouth of an adult female Nile tilapia. Upon collection, the eggs were immersed in 30% hydrogen peroxide (Merck-Millipore, Amsterdam, The Netherlands) and diluted in autoclaved synthetic freshwater (ASF) with a final active peroxide concentration of 2 g L^−1^ for 10 min at 26 ± 1 °C. The synthetic freshwater contained 96 mg L^−1^ NaHCO_3_, 60 mg L^−1^ CaSO_4_·2H_2_O, 60 mg L^−1^ MgSO_4_ and 4 mg L^−1^ KCl in nanopure water^42^. The eggs were subsequently washed four times with 250 mL of 0.2 μm-filtered ASF at 26 ± 1 °C to remove loose bacteria and damaged eggs. Twenty-four hours later, a second disinfection was conducted using 100 mL of NaClO (14%) in 1 L of ASF, following the same immersion protocol as described for day 1. During the immersion, the beakers were occasionally shaken to ensure the optimal penetration of disinfectant into the eggs. This disinfection method was applicable only prior to egg hatching, as this strategy was considered unsafe or lethal for larvae^41^. On day 3, as soon as the eggs began to hatch, the disinfection process was conducted by the immersion of eggs/larvae into 1 L of ASF containing 100 mg/L Bronopol (2-bromo-2-nitropropane-1,3-diol; Sigma-Aldrich 13,470-8, Zwijndrecht, The Netherlands) for 30 min. Bronopol disinfection was performed daily from day 3 to 7 during the axenic stage. All disinfection procedures were performed in a laminar flow hood, and all equipment and tools were autoclaved prior to use.

Following disinfection on day 3, the hatched eggs were aseptically distributed into 2-L sterile glass bottles (Duran GL45) containing 500 mL axenic incubation medium (Table S1) and incubated at a density of 300 eggs L^−1^. Air was provided to the bottles through a filter (0.25 μm, Whatman) from a single port safety cap (Duran DG), and the bottles were placed in a moving water-bath (Julabo SW23, 110 rpm at 27 °C). During the axenic period (day 1 to 7), the larvae were not fed.

On day 8, a total of 144 axenic larvae were equally distributed into the two probiotic chambers and exposed to the probiotic bacteria, whereas 120 larvae were equally distributed into the three AS aquaria and exposed to conventional (control) rearing conditions (see below for details).

### Probiotic conditions

To rear the larvae during the probiotic stage a custom-made cabinet was used (Figure S1). The larvae were reared in two custom-made 2-L glass chambers installed inside the cabinet. The air in the cabinet was pressurized, and the internal surfaces were sterilized at hourly intervals through UV irradiation. The chambers were water-heated at 27 ± 1 °C using the space available within the double-layered outer glass wall. Air was supplied through 0.25-μm syringe filters (Whatman) placed on single port safety caps (Duran DG). Larvae collection and water replacement were conducted through a bottom outlet valve (Figure S1). The larvae were sieved and washed with ASF daily and subsequently externally disinfected with Bronopol solution (100 μl/L) for 30 min (see axenic stage disinfection from day 3 onwards). The incubation chambers were replaced daily with autoclaved chambers.

The probiotic strain, *B. subtilis* (Microbiologics 0269P, Mijdrecht, The Netherlands), was grown for 24 h on E-type agar containing 15 g L^−1^ bacteriological agar type E, 10 g L^−1^ Tryptone, 5 g L^−1^ NaCl, and 5 g L^−1^ yeast extract powder in 1 L of demineralized water. The bacterial colonies were further cultured in 100 mL of liquid medium containing 7 g L^−1^ K_2_HPO_4_, 2.0 g L^−1^ KH_2_PO_4_, 1.0 g L^−1^ (NH_4_)_2_SO_4_, 1.0 g L^−1^ glucose, 0.5 g L^−1^ sodium citrate, and 0.1 g L^−1^ MgSO_4_.7H_2_O in demineralized water for another 24 h. Prior to use, the cultures were centrifuged for 7 min (8000 × *g*) to pellet the *B. subtilis*. The supernatant was discarded, and the cell density was adjusted to 1 × 10^7^ cfu mL^−1^ with 1.5 L ASF water as spectrophotometrically determined by optical density (OD_600_) (Bio-Rad SmartSpec 3000). In the probiotic chambers, the water containing *B. subtilis* was replaced daily.

### Active suspension tanks

Six 20-L aquaria, each connected to a separate 120-L active suspension tank (AST), were used for rearing the larvae under conventional conditions. One month prior to the experiment, 10 adult tilapias were stocked per AST to initiate microbial growth. Prior to the addition of the axenic larvae, adult tilapias were removed, and the water from all six ASTs was mixed and re-distributed over the tanks. Water and suspended solids in the ASTs were constantly mixed and aerated, while the temperature was maintained at 27 °C. Total ammonia nitrogen (TAN-N), nitrite (NO_2_^−^-N), nitrate (NO_3_^−^-N), dissolved oxygen (DO), pH and temperature (°C) were monitored daily in each AST.

During their first week in the ASTs (day 8 to 14), the larvae from the control treatment and in the probiotic chambers were not fed (Fig. 1). On day 14, the larvae from both probiotic chambers were mixed and redistributed over the three active suspension tanks (40 larvae/tank). Thereafter, the larvae from both treatments were fed daily to apparent satiation (30 min) at 09:00, 12:30 and 16:00 with a commercial crumble larval feed (Skretting Gemma Wean M0.5, 300–500 μm, 58% crude protein, 17% crude fat, 10% ash, 0.6% fibre and 1.3% phosphorus). The feed was divided into daily portions of 4 g in 15-mL Falcon tubes and subsequently sterilized with cobalt-60 gamma irradiation at 25 kGy (Synergy Health, Ede, The Netherlands) prior to the experiment to minimize the viable microbial load entering with the feed, and the 16 S ribosomal RNA (rRNA) gene-targeted PCR using DNA extracted from irradiated feed did not yield any products.

### Verification of axenic and probiotic conditions by cultivation

During the axenic and probiotic stages, daily samples of the culture medium, eggs and media/water were monitored for the presence of viable bacterial cells. The eggs, larvae and 1 mL of water were separately added into 10 mL of liquid medium (as previously described for *B. subtilis*) and incubated at 37 °C for 24 h. The next day, the samples were streaked onto 2YT agar (Biotrading K604P090KP, Mijdrecht, The Netherlands) and incubated for 24 h at 37 °C. At 48 h after inoculation, the agar plates were visually assessed for microbial growth.

### Sampling of gut and water for bacterial community profiling

For each of the aquaria/chambers, the gut samples from four larvae were collected on days 14, 21 and 28 (Table S2, sample meta-data), and the water was also sampled from each aquarium/chamber after filtering 1 L of water through 0.45- and 0.2-μm membrane filters (Millipore HAWP-04700 and Millipore GTTP-04700). All samples were frozen in liquid nitrogen and stored at −80 °C until further analysis. All gut and water samples were stored and individually analysed. The detailed protocols on gut and water sampling are described in Giatsis *et al*.^43^.

### Bacterial community profiling

DNA was extracted from gut samples using the DNeasy Blood & Tissue Kit (Qiagen, Venlo, The Netherlands) according to the manufacturer’s protocol, with some modifications^43^. For DNA extraction from the water samples, the FastDNA SPIN kit for soil (MP Biomedicals, Ohio, USA) was used. The DNA concentrations were measured with a NanoDrop ND-1000 spectrophotometer (NanoDrop^®^ Technologies, Wilmington, DE), and the DNA samples were stored at −80 °C until further use. Detailed protocols on gut and water DNA extraction are described elsewhere^43^.

For 16S rRNA gene-based microbial composition profiling, barcoded amplicons from the V4 region of 16S rRNA genes were generated by PCR using the 515F and 806R primers^44^. Seventy different barcodes were used per library, in which the forward and reverse primer of one sample always carried the same barcode. The primer sequence and barcode were separated by a 2-nucleotide linker sequence (GA for 515F and CG for 806R). The extracted DNA was diluted to a concentration of 20 ng μL^−1^ based on NanoDrop (NanoDrop Technologies, Wilmington, DE) spectrophotometric readings. The PCR conditions, DNA purification and library preparations were performed according to Giatsis *et al*.^24^. The nucleotide sequences were generated using an Illumina HiSeq 2000 sequencer at GATC-Biotech, Konstanz, Germany. Raw sequence data were deposited into the Sequence Read Archive (SRA) at the NCBI under accession number SRP062681.

NG-Tax, an in-house pipeline, was used for the analysis of the 16 S rRNA gene sequencing data^45^. Briefly, paired-end libraries were filtered to contain only read pairs with perfectly matching barcodes, and these barcodes were used to separate reads according to sample. The operational taxonomic units (OTUs) were assigned and classified using an open reference approach and a customized SILVA 16 S rRNA gene reference database^46^.

### Data handling and statistical analysis

The Bray Curtis dissimilarity was calculated based on square root-transformed relative abundance data. Principal coordinate analysis (PCoA) was performed to represent the samples in a low dimensional space; thus, the relative distances of all points represent the relative dissimilarities of the samples according to the Bray Curtis index. All statistical analyses were performed using the multivariate statistical software package Primer V7 (Primer-E Ltd, Plymouth, UK). BLAST searches were used to identify the closest relatives of selected OTUs (members of the genus *Bacillus*)^47^. Multiple sequence alignments of the sequences were performed in ClustalW2-Phylogeny using neighbour-joining as the clustering method, and the corresponding Newick tree file was visualized using a phylogram constructed in Treedyn^48^.

## Results

### Axenic stage

In total, 380 fertilized eggs were available at the start of the experiment. By the end of the hatching period, 304 larvae (80% of all eggs) successfully hatched. On day 7, 120 of the axenic larvae were equally distributed into active suspension tanks C1, C2 and C3 (named as “control treatment”), whereas the remaining larvae were exposed to a high load of *B. subtilis* in chambers P-CH1 and P-CH2 (named “probiotic treatment”) for one week.

Medium and egg samples cultured on agar plates showed no proliferation of microbes throughout the axenic period, and 16S rRNA gene-targeted PCR using DNA extracted from washed and antibiotic-treated eggs and larvae yielded no products, confirming the axenic conditions.

### Probiotic stage

**Figure 2:**
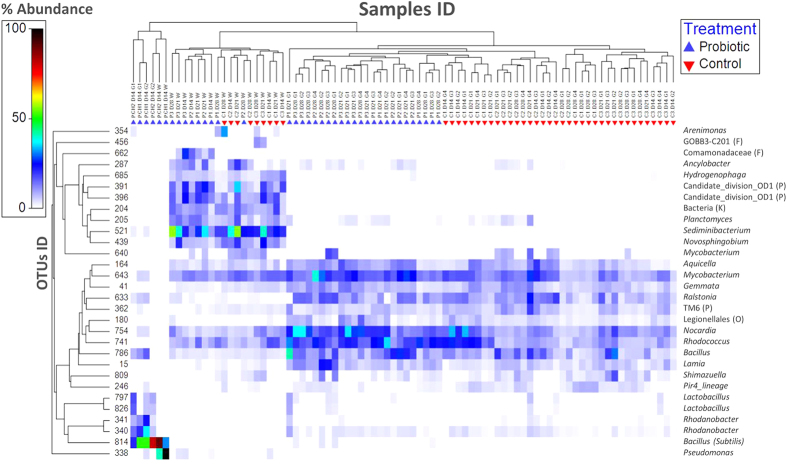
Heatmap of the 30 most predominant OTUs among all water and gut samples. Each column in the heatmap represents a sample, and each row represents an OTU. The OTUs were clustered based on group average, as groups of OTUs better define sample clusters. Only the first thirty OTUs contributing most to these clusters are displayed. The samples were clustered according to the unweighted pair group method with arithmetic mean (UPGMA) hierarchical clustering on the basis of Bray Curtis dissimilarity and based on the complete OTU dataset. The colours are proportional to the increasing percent relative OTU abundance (from white: lower, to red: higher) within each sample. P1-3 and C1-3: Replicate active suspension tanks 1-3 of the probiotic and control treatment, respectively. D14, 21 and 28: Experimental days 14, 21 and 28. G1-4: The number of replicate gut samples from each tank. G and W: Gut and water samples, respectively. P-CH1 and 2 (probiotic chamber 1 and 2) indicate that water samples were obtained from the probiotic chamber at the end of probiotic period (day 14). Taxonomy (right column) indicates the genus of each OTU ID (left column) unless otherwise stated (i.e., order (O), family (F) and phylum (P)).

The 16S rRNA profiling of water microbiota from chambers P-CH1 and P-CH2 on day 14 confirmed the presence of the probiotic strain in both chambers albeit at different relative abundances. P-CH2 was dominated with *Bacillus*, whereas P-CH1 was dominated with *Pseudomonas* (Fig. 2). In both chambers, several OTUs belonging to the genus *Bacillus* were present; however, the most abundant *Bacillus* OTU (OTU 814) had 100% sequence identity with the added probiotic strain of *B. subtilis* (Figure S2)*. Pseudomonas* OTU 338 was present in the water of both chambers at a relative abundance of >20%.

**Figure 3:**
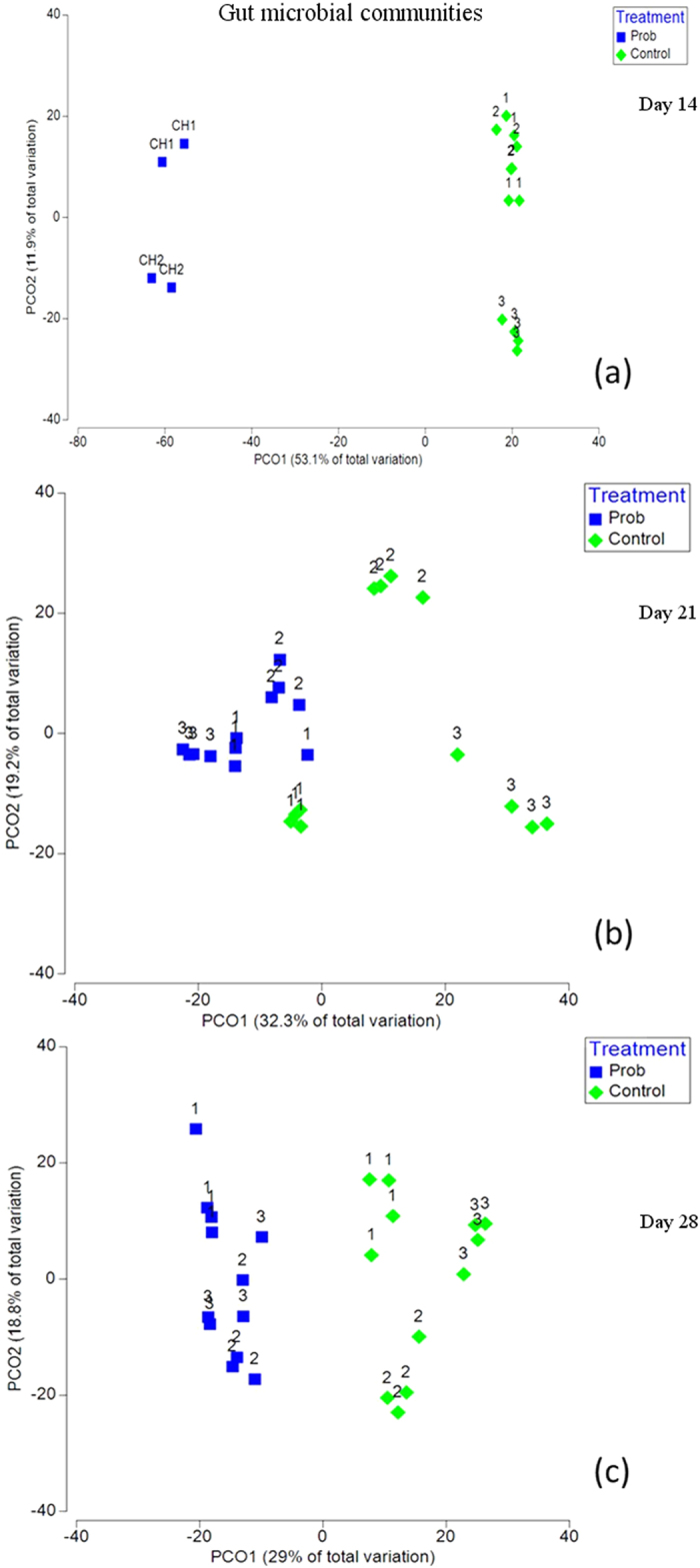
Principal coordinate analysis (PCoA) of the gut microbial communities based on the square root-transformed relative abundance data of OTUs. The relative distances of all points represent the relative dissimilarities of the samples according to the Bray Curtis index. Plots (**a–c**): ordinations of all gut samples from probiotic and control treatment from days 14, 21 and 28, respectively. The percentage of total variation explained by each PCo axis is shown in the parentheses.

Subsequent analyses of the gut samples from larvae raised in the probiotic chambers showed that *B. subtilis* was among the most dominant species, regardless of the observed differences in the relative abundance of *B. subtilis* in the corresponding water samples. At the end of the probiotic treatment (day 14), *B. subtilis* accounted for approximately half of all bacteria in the gut (average relative abundance). A comparison between the gut samples from control (C1, C2 and C3 tanks) and probiotic treatments (P-CH1 and P-CH2 chambers) indicated a clear difference in the composition of the gut microbiota (Fig. 3a). This difference reflected, in part, the high relative abundance of *B. subtilis* in the gut of larvae from the probiotic treatment (and the absence of these bacteria from the control), according to the SIMPER analysis results (contribution: 25%). Other discriminant OTUs were members of the genera *Nocardia, Mycobacterium, Rhodococcus, Rhodanobacter and Halomonas* (Table S3).

### Active suspension stage

At the end of the probiotic stage (day 14), larvae from the probiotic chambers were transferred to active suspension tanks (P1, P2 and P3). One week after exposure to conventional rearing conditions (day 21), their gut microbiota was significantly different from that of the control treatment larvae, which had been acclimated to non-sterile conditions for one week longer (Fig. 3b and Table S4). The replicate aquaria of the control treatment were significantly more dispersed than those of the probiotic treatment according to multivariate permutation dispersion (P_perm_: 0.011) (Table S5). However, the observed lower dispersion within the probiotic treatment no longer reflected the presence of *B. subtilis* in the gut, as the relative abundance of these bacteria at day 21 was below detection level. The most predominant OTUs in the probiotic treatment were members of the genera *Bacillus*, *Rhodococcus*, *Nocardia*, *Mycobacterium, Ralstonia and Aquicella* (Fig. 2). These taxa were also among the most predominant bacteria observed in the gut of the control treatment on day 21 but at different relative abundances. One *Bacillus*-affiliated OTU (OTU 786) was present in all gut samples of both treatments on day 21, with an average relative abundance of 4.1% (SD ± 2.3). BLAST analysis showed that this bacterium was a different species than the administered strain, as its 16S rRNA gene sequence was only 94% identical to that of the probiotic strain of *B. subtilis* (Figure S2).

A comparison of the gut microbiota on day 28 showed significantly different communities in the two treatments (Fig. 3c and Table S4). As on day 21, the probiotic strain remained below the detection level. The most predominant genera at day 28 in both treatments were similar to those observed at day 21, albeit at different relative abundances. The within-treatment variability of replicate tanks was no longer significantly different (P_perm_: 0.121) between control and probiotic treatments (Table S5). Notably, the homogeneity of dispersion is a precondition to accurately interpret, but not perform, a PERMANOVA. On day 21, significant differences in the gut communities were detected in both PERMDISP (dispersion) and PERMANOVA (location). Thus, to uncover the nature of the differences among groups, the results are also discussed with respect to the average within- and between-group dissimilarities and the position of the samples from different groups in the PCoA.

### Succession of water and gut microbiota

**Figure 4:**
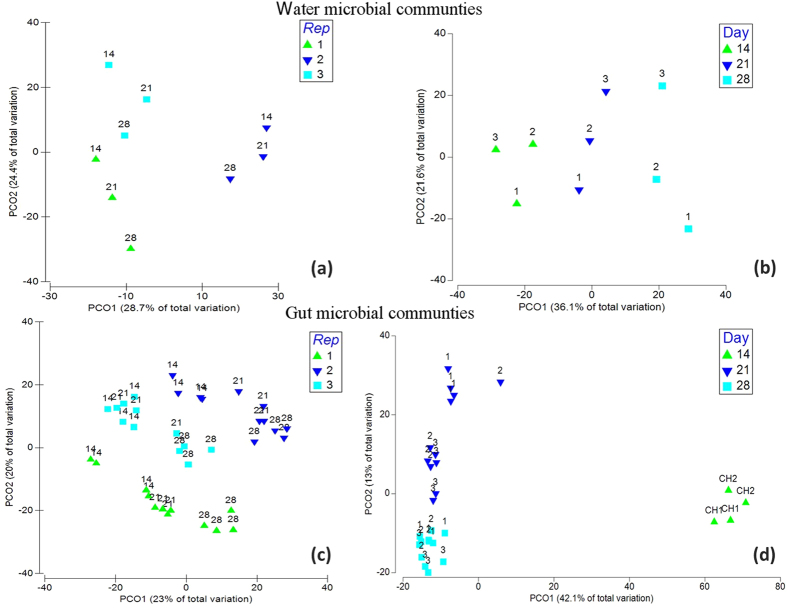
Principal coordinate analysis (PCoA) of microbial communities in the gut and water samples of the control and probiotic treatments. The plots are based on the square root-transformed relative abundance data for the OTUs. The relative distances of all points represent the relative dissimilarities of the samples according to the Bray Curtis index. Ordination plots of water (**a,b**) and gut (**c,d**) microbial communities from the control (**a,c**) and probiotic treatments (**b,d**), respectively. The numbers 14, 21 and 28 indicate the three experimental days that the samples were collected. Rep 1-3: Replicate tank 1 to 3. The percentage of total variation explained by each PCo axis is shown in the parentheses.

The effect of time on the water and gut microbiota composition was evaluated by comparing the profiles on days 14, 21 and 28. The water microbiota in the tanks was not significantly different between the two treatments on any of the sampling days, but a different pattern was revealed over time (Table S6), i.e., water microbial communities from the control treatment clearly clustered by tank, whereas this pattern was not observed in the probiotic treatment (Fig. 4a,b). *Sediminibacterium* (OTU 521) was the most abundant genus in the water of both treatments on days 21 and 28, with average relative abundances between 18% and 35%, respectively (Fig. 2).

Regarding the gut samples, the cluster analysis revealed clearly different patterns between treatments. In the control treatment, the variability of the gut microbial communities was higher between tanks than between days (Fig. 4c), consistent with the pattern observed in the water (Fig. 4a). In the probiotic treatment, the tank effect was not as clear as in the case of the control (Fig. 4d).

## Discussion

Probiotics have been widely applied in aquaculture for many years. However, assessing the effectiveness of their use is problematic because the probiotic strains residing in the gut transiently and rapidly fall below detection limits. This effect most likely reflects the low survival and proliferation rate of probiotics in the fish gut^7^,^14^. For prolonged gut colonization by probiotics, it is paramount to understand the principles governing microbial community assembly and the persistence of specific populations.

In the present study, we attempted to enhance the colonization success of the probiotics in a “virgin” gut ecosystem by maintaining the larvae in axenic conditions prior to exposure to a probiotic strain. The results indicated that *B. subtilis* was present in the waters of both probiotic chambers, albeit at different relative abundances. In the first chamber, *B. subtilis* was the most dominant water OTU; in the second chamber, populations belonging to the genus *Pseudomonas* were present at a higher relative abundance, although the same amount of *B. subtilis* was added daily to both chambers. Apparently, we were not successful in maintaining the gnotobiotic conditions of the water, as both chambers were contaminated with *Pseudomonas*.

However, regardless of the presence of *Pseudomonas* in the water of both P-CH, *Pseudomonas* was barely detected in the gut at that time point. These results demonstrated that (a) *B. subtilis* can be successfully transferred to the gut through water, and (b) *Pseudomonas* cannot be successfully transferred to the gut irrespective of its abundance in the source water. Members of the genus *Pseudomonas* are non-sporulating, aerobic Gram-negative rods observed in a wide range of terrestrial and aquatic environments in addition to plant and animal-associated ecosystems. Consistent with this broad environmental distribution, these bacteria exhibit metabolic versatility^49^,^50^. In fish, *Pseudomonas* is commonly observed in the faeces and gut of both salt and fresh water species^51^,^52^. Various phenomena, such as the competitive exclusion of *Pseudomonas* by *B. subtilis*, differences in the ecological preference/adaptability of the two species in the gut and host selectivity for *Bacillus* but not *Pseudomonas*, may have played a role in the recovery of *B. subtilis* but not *Pseudomonas* from the guts of tilapia larvae during probiotic treatment.

In the present study, *B. subtilis* was only transiently detected and thus was not included in the stable larval microbiota. One week after the larvae were exposed to conventional aquaculture conditions (day 21), the abundance of *B. subtilis* was already below detection level. This finding underscores the challenge of successfully colonizing the fish gut with a probiotic strain. The presence of this strain in the gut can be expected only until a few days after probiotic discontinuation, consistent with previous studies reporting that probiotic strains added through water or feed could be detected in the guts of fish and shrimp for only a few days after discontinuing application of the probiotic^16^,^17^,^32^,^53^,^54^. In a recent study, Standen *et al*.^55^ observed that the presence of a multi-species probiotic containing *Lactobacillus reuteri*, *Bacillus subtilis*, *Enterococcus faecium* and *Pediococcus acidilactici* gradually declined after probiotic cessation. However, the detection of each probiotic strain in the gut varied between 6 and 18 days after reverting to the control diet, suggesting that the persistence of probiotics in the gut is species-specific. Furthermore, the probiotic supplemented feed was administered to adult tilapia for eight weeks, whereas here, *B. subtilis* was administered to axenic tilapia larvae for one week. The dosage and duration of supplementation and the selection of the probiotic strain/s might influence colonization success, and the persistence of the probiotic might also depend on the developmental state of the animal^49^,^56^,^57^.

In the present study, the development of gut microbial communities in the two treatments revealed different patterns. The gut microbiota in the control treatment were more affected by spatial (tank) rather than temporal differences (time), i.e., the samples clustered according to replicate tank rather than sampling day (Fig. 4c). Interestingly, the spatiotemporal patterns observed in gut bacterial communities were also observed in the water microbiota of the control treatment. In a previous study on tilapia larvae, we observed that tank replication determined the inter-individual variation of gut microbiota (Giatsis *et al*.^43^). Here, this finding was differently applied for each treatment. This difference could be associated with the initial contact of larvae with the probiotic strain. At the time larvae from the probiotic treatment were introduced to conventional conditions, their guts were already colonized with certain bacteria (primarily *B. subtilis*), whereas the larvae from the control treatment were introduced to conventional conditions while their guts were germ-free.

The successful transfer via water and the high relative abundance of the probiotic strain in the gut indicate that it is conceivable to inoculate the gut community with bacteria during early gut development. At the end of the probiotic stage, four gut samples were collected from both probiotic chambers. The observed inter-individual variation in the abundance of the probiotic strain in the gut suggests that the results should be interpreted with caution. Studies on the use of probiotics in humans and animals have also reported high inter-individual variation, even for identically treated groups^58^,^59^,^60^,^61^. The abundance of a probiotic strain in the gut or faeces is neither clear-cut proof of successful probiotic use nor evidence of probiosis, primarily reflecting the difficulty in establishing the precise relationships between the health benefits and the presence and/or relative abundance of a specific microbe (except for specific pathogens)^57^. Notably, inter-individual variation could certainly mask treatment effects by either type-I or type-II errors. Thus, more data points (higher statistical power) should be included in future studies to verify whether the observed correlations are maintained.

**Figure 5:**
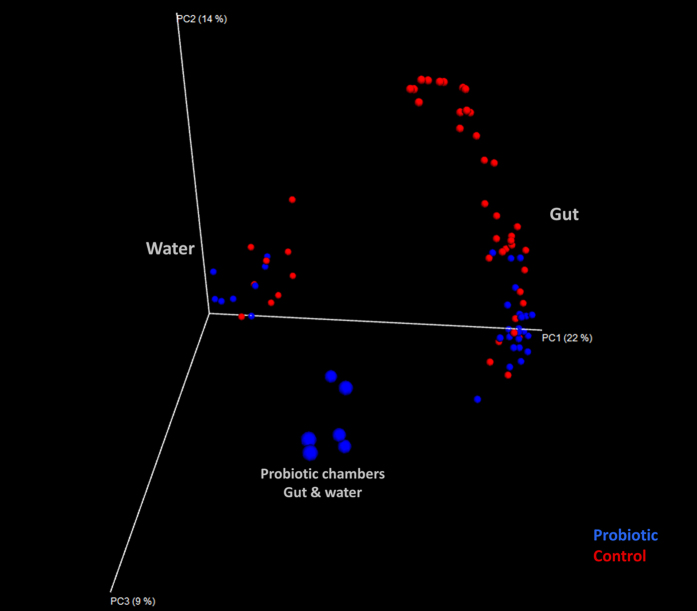
Principal coordinate analysis (PCoA) of the gut (right) and water (left) microbial communities from all sampling days (14, 21 and 28) and treatments (probiotic and control). The blue spheres along the PC3 (centre) correspond to water and gut samples from the probiotic chambers on day 14. This analysis was based on the square root-transformed relative abundance data of the OTUs. The relative distances of all points represent the relative dissimilarities of the samples according to the Bray Curtis index. The percentage of total variation explained by each of the three first PCo axes is shown in the parentheses.

The observed low persistence of the probiotic strain in the gut could indicate a lack of ecological preference or adaptability of the probiotic strain in the gut and/or host selectivity against the probiotic. Nevertheless, the gut communities remained different between treatments, even after discontinuation of the probiotic and despite receiving the same diet and living in water containing similar microbial profiles (Fig. 5). It is doubtful (although there is no clear-cut evidence) that the presence of *Pseudomonas* in the probiotic chambers induced the observed probiotic legacy effects, as Pseudomonas, although present in the water, was nearly absent from the larval gut. It is more likely that the initial presence of *B. subtilis* led to a different sequence of events of bacterial colonization, reflecting the synergistic or antagonistic interactions between the bacteria already present and/or other bacteria entering the gut. It is also likely that the transition from axenic to either conventional or probiotic conditions differentially modulated the immune response and mucosal innate immunity of the larvae. The responses of IgA, cytokine production and the development of mucosal T-regulatory cells were likely reduced in germ-free animals through the activation of TLR-dependent pathways^62^,^63^,^64^. TLR9 was expressed on the colonic apical surface in wild type but not germ-free mice^65^. These results demonstrated that the gut microbiota alters the way the host reacts to infectious stimuli or particular bacterial taxa^66^ entering the gut, and this difference could also be the case in the present study. Differences in the initial priming of the immune system in the probiotic group are certainly among the potential mechanisms^67^,^68^.

After the discontinuation of probiotic administration, differences in the gut microbiota between treatments primarily reflected differences in the relative abundance of the genera *Nocardia, Mycobacterium, Rhodococcus, Rhodanobacter, Halomonas* and *Ralstonia*. Most of these genera have been identified in previous studies on tilapia larvae and other fish species. The genus *Rhodococcus* has been reported in the guts of tilapia, sole, red rock fish, Norwegian mackerel, USA smelt, rainbow trout and shrimp^24^,^69^,^70^,^71^,^72^, and the genus *Nocardia* has been reported in the guts of tilapia^24^,^73^ and Atlantic salmon^74^. *Ralstonia* has been observed in the guts of seabass^75^, rainbow trout^22^,^69^,^76^, yellow catfish^77^, zebrafish^78^ and shrimp^79^. Furthermore, members of the genus *Halomonas* have been reported in the guts of Arctic charr^80^, Atlantic cod^81^, Midas cichlids^82^, queen conch^83^ and Artemia brine shrimp^84^,^85^. These findings indicate that some of the predominant genera observed in the present study could represent common members of the gut microbiota of tilapia larvae or fish in general, suggesting that (a) host-specificity for particular microbial taxa is modulated by selective pressures within the host gut, and (b) these taxa are involved in major metabolic functions in the fish gut. Host-selective capabilities have been revealed in axenic zebrafish (*Danio rerio*) that received a faecal transplant derived from mice. The implanted mouse community subsequently shifted towards a state resembling a native zebrafish community^21^. In addition, zebrafish originating from the wild shared a core gut microbiota with those reared in captivity, demonstrating a host-specific microbial community in the gut^86^. The observed differences in the abundance of these genera within and between studies could reflect a certain degree of influence of the environmental microbiota (i.e., available bacteria, including the probiotic strain used in the present study), community-level interactions and dietary interventions, underlying powerful organizing principles in community composition.

To what extent post-treatment gut microbial uniformity or distinctness reflects a sustained effect of the probiotic remains unknown. We suggest that future studies focus on the long-term effects of probiotic legacy during the early developmental stages of animals. To observe a general phenomenon, future experiments are needed to determine how this effect compares with that of antibiotic or dietary interventions. It has been suggested that legacy effects in humans play a role in defining the microbial structure during early life stages, and these effects can be minimized based on the diet of the host^87^. If this idea also applies to animals, then the early administration of a probiotic strain, accompanied by continuous prebiotic administration, could further extend probiotic residence in the gut, even after its discontinuation.

## Additional Information

**How to cite this article**: Giatsis, C. *et al*. Probiotic legacy effects on gut microbial assembly in tilapia larvae. *Sci. Rep*. **6**, 33965; doi: 10.1038/srep33965 (2016).

## Supplementary Material

Supplementary Information
