# Supplementary material for: Probiotic legacy effects on gut microbial assembly in tilapia larvae
Source: Sci Rep. 2016 Sep 27;6:33965. doi: 10.1038/srep33965 (PMC5037425; doi:10.1038/srep33965)
Supplement: Supplementary Information [file srep33965-s1.pdf]

Probiotic legacy effects on gut microbial assembly in tilapia larvae

Christos Giatsis[1]*, Detmer Sipkema[2], Javier Ramiro-Garcia[2,3,4], Gianina M. Bacanu[2], Jason Abernathy[5], Johan Verreth[1], Hauke Smidt[2], and Marc Verdegem[1]

[1] Aquaculture and Fisheries Group, Wageningen University, De Elst 1, 6708 WD Wageningen, The Netherlands

[2] Laboratory of Microbiology, Wageningen University, Stippeneng 4, 6708 WE Wageningen, The Netherlands

[3] Laboratory of System and Synthetic Biology, Stippeneng 4, Wageningen 6708 WE, The Netherlands

[4] TI Food and Nutrition (TIFN) P.O. Box 557, 6700 AN, Wageningen 6703 HB, The Netherlands

[5] USDA-ARS, Hagerman Fish Culture Experiment Station, 3059F National Fish Hatchery Road, Hagerman, Idaho 83332, USA

Table S1.
Antibiotics and antifungals used for the preparation of the incubation medium. All components were diluted in autoclaved synthetic freshwater (ASF) to the concentration indicated in the table. Original protocol has been obtained by Situmorang et al. (2014) and modifications have been applied regarding the addition of Gentamycin in the incubation medium.

Name	Concentration	Dosage	Description	Source
Kanamycin Sulphate	Sigma T7783	1 ml L^{-1}	Broad spectrum antibiotic	(Situmorang et al. 2014)
Rifampicin	Sigma K1377	1 ml L^{-1}	Broad spectrum antibiotic	(Situmorang et al. 2014)
Ampicillin sodium salt	Sigma A0166	1 ml L^{-1}	Broad spectrum antibiotic	(Situmorang et al. 2014)
Trimethoprim	Sigma 89307	1 ml L^{-1}	Bacteriostatic antibiotic	(Situmorang et al. 2014)
Gentamicin	Sigma G1397	50 μl L^{-1}	Gram-negative antibiotic	Rosco NeoSensitabs
Amphotericin B	Sigma A9528	100 μl L^{-1}	Polyene antifungal	(Situmorang et al. 2014)
Fluorescent Brightener 28	Sigma F3543	2.5 ml L^{-1}	Antifungal	(Situmorang et al. 2014)

Table S2. Meta-data of gut (G) and water (W) samples from the control (C) and probiotic (P) treatment. Microbial communities were characterized on day (D) 14, 21 & 28 in the probiotic chamber (P-CH) and/or the active suspension tanks. Two replicate probiotic chambers and three replicate active suspension tanks were sampled per treatment (C1-3 & P1-3) from which a water and four replicate gut samples (G1-4) were analysed.

No	Sample_ID	Treatment	Culture system	Biome	Day	Tank	Replicate
1	P-CH1.D14.W	Probiotic	Probiotic chamber	Water	14	1	-
2	P-CH2.D14.W	Probiotic	Probiotic chamber	Water	14	2	-
3	C1.D14.W	Control	Active suspension	Water	14	1	-
4	C2.D14.W	Control	Active suspension	Water	14	2	-
5	C3.D14.W	Control	Active suspension	Water	14	3	-
6	P1.D14.W	Probiotic	Active suspension	Water	14	1	-
7	P2.D14.W	Probiotic	Active suspension	Water	14	2	-
8	P3.D14.W	Probiotic	Active suspension	Water	14	3	-
10	C1.D21.W	Control	Active suspension	Water	21	1	-
11	C2.D21.W	Control	Active suspension	Water	21	2	-
12	C3.D21.W	Control	Active suspension	Water	21	3	-
13	P1.D21.W	Probiotic	Active suspension	Water	21	1	-
14	P2.D21.W	Probiotic	Active suspension	Water	21	2	-
15	P3.D21.W	Probiotic	Active suspension	Water	21	3	-
17	C1.D28.W	Control	Active suspension	Water	28	1	-
18	C2.D28.W	Control	Active suspension	Water	28	2	-
19	C3.D28.W	Control	Active suspension	Water	28	3	-
20	P1.D28.W	Probiotic	Active suspension	Water	28	1	-
21	P2.D28.W	Probiotic	Active suspension	Water	28	2	-
22	P3.D28.W	Probiotic	Active suspension	Water	28	3	-
24	C1.D14.G1	Control	Active suspension	Whole gut	14	1	1
25	C1.D14.G2	Control	Active suspension	Whole gut	14	1	2
26	C1.D14.G3	Control	Active suspension	Whole gut	14	1	3
27	C1.D14.G4	Control	Active suspension	Whole gut	14	1	4
28	C2.D14.G1	Control	Active suspension	Whole gut	14	2	1
29	C2.D14.G2	Control	Active suspension	Whole gut	14	2	2
30	C2.D14.G3	Control	Active suspension	Whole gut	14	2	3
31	C2.D14.G4	Control	Active suspension	Whole gut	14	2	4
32	C3.D14.G1	Control	Active suspension	Whole gut	14	3	1
33	C3.D14.G2	Control	Active suspension	Whole gut	14	3	2
34	C3.D14.G3	Control	Active suspension	Whole gut	14	3	3
35	C3.D14.G4	Control	Active suspension	Whole gut	14	3	4
36	C1.D21.G1	Control	Active suspension	Whole gut	21	1	1

37	C1.D21.G2	Control	Active suspension	Whole gut	21	1	2
38	C1.D21.G3	Control	Active suspension	Whole gut	21	1	3
39	C1.D21.G4	Control	Active suspension	Whole gut	21	1	4
40	C2.D21.G1	Control	Active suspension	Whole gut	21	2	1
41	C2.D21.G2	Control	Active suspension	Whole gut	21	2	2
42	C2.D21.G3	Control	Active suspension	Whole gut	21	2	3
43	C2.D21.G4	Control	Active suspension	Whole gut	21	2	4
44	C3.D21.G1	Control	Active suspension	Whole gut	21	3	1
45	C3.D21.G2	Control	Active suspension	Whole gut	21	3	2
46	C3.D21.G3	Control	Active suspension	Whole gut	21	3	3
47	C3.D21.G4	Control	Active suspension	Whole gut	21	3	4
48	C1.D28.G1	Control	Active suspension	Whole gut	28	1	1
49	C1.D28.G2	Control	Active suspension	Whole gut	28	1	2
50	C1.D28.G3	Control	Active suspension	Whole gut	28	1	3
51	C1.D28.G4	Control	Active suspension	Whole gut	28	1	4
52	C2.D28.G1	Control	Active suspension	Whole gut	28	2	1
53	C2.D28.G2	Control	Active suspension	Whole gut	28	2	2
54	C2.D28.G3	Control	Active suspension	Whole gut	28	2	3
55	C2.D28.G4	Control	Active suspension	Whole gut	28	2	4
56	C3.D28.G1	Control	Active suspension	Whole gut	28	3	1
57	C3.D28.G2	Control	Active suspension	Whole gut	28	3	2
58	C3.D28.G3	Control	Active suspension	Whole gut	28	3	3
59	C3.D28.G4	Control	Active suspension	Whole gut	28	3	4
60	P1.D14.G1	Probiotic	Probiotic chamber	Whole gut	14	1	1
61	P1.D14.G2	Probiotic	Probiotic chamber	Whole gut	14	1	2
62	P2.D14.G1	Probiotic	Probiotic chamber	Whole gut	14	2	3
63	P2.D14.G2	Probiotic	Probiotic chamber	Whole gut	14	2	4
64	P1.D21.G1	Probiotic	Active suspension	Whole gut	21	1	1
65	P1.D21.G2	Probiotic	Active suspension	Whole gut	21	1	2
66	P1.D21.G3	Probiotic	Active suspension	Whole gut	21	1	3
67	P1.D21.G4	Probiotic	Active suspension	Whole gut	21	1	4
68	P2.D21.G1	Probiotic	Active suspension	Whole gut	21	2	1
69	P2.D21.G2	Probiotic	Active suspension	Whole gut	21	2	2
70	P2.D21.G3	Probiotic	Active suspension	Whole gut	21	2	3
71	P2.D21.G4	Probiotic	Active suspension	Whole gut	21	2	4
72	P3.D21.G1	Probiotic	Active suspension	Whole gut	21	3	1
73	P3.D21.G2	Probiotic	Active suspension	Whole gut	21	3	2
74	P3.D21.G3	Probiotic	Active suspension	Whole gut	21	3	3
75	P3.D21.G4	Probiotic	Active suspension	Whole gut	21	3	4
80	P1.D28.G1	Probiotic	Active suspension	Whole gut	28	1	1
81	P1.D28.G2	Probiotic	Active suspension	Whole gut	28	1	2

82	P1.D28.G3	Probiotic	Active suspension	Whole gut	28	1	3
83	P1.D28.G4	Probiotic	Active suspension	Whole gut	28	1	4
84	P2.D28.G1	Probiotic	Active suspension	Whole gut	28	2	1
85	P2.D28.G2	Probiotic	Active suspension	Whole gut	28	2	2
86	P2.D28.G3	Probiotic	Active suspension	Whole gut	28	2	3
87	P2.D28.G4	Probiotic	Active suspension	Whole gut	28	2	4
88	P3.D28.G1	Probiotic	Active suspension	Whole gut	28	3	1
89	P3.D28.G2	Probiotic	Active suspension	Whole gut	28	3	2
90	P3.D28.G3	Probiotic	Active suspension	Whole gut	28	3	3
91	P3.D28.G4	Probiotic	Active suspension	Whole gut	28	3	4

Table S3. Similarity percentages (SIMPER) analysis of gut microbiota on day 14. Table indicates the foremost three characteristic OTUs from each group contributing to the discrimination between the control and probiotic treatment. Contribution values indicate the importance of each OTU (percentage) in increasing Bray Curtis dissimilarity between the two treatments.

Similarity Percentages - species contributions
Data type: Abundance
Resemblance: Bray-Curtis dissimilarity

Groups Control & Probiotic
Average dissimilarity = 96.82

Genus (OTUs)	Group Control Av. Abundance	Group Probiotic Av. Abundance	Av. Dissimilarity	Contrib%	Cum.%
**Bacillus (814)**	0	47.95	23.97	24.76	24.76
**Nocardia (754)**	12.45	0.12	6.16	6.37	31.13
**Mycobacterium (643)**	11.53	0.18	5.67	5.86	36.99
**Rhodococcus (741)**	9.21	0	4.61	4.76	41.75
**Rhodanobacter (340)**	0.34	8.2	3.94	4.07	45.81
**Halomonas (496)**	0.25	3.6	1.69	1.74	47.55

Table S4. Permutational MANOVA test and pairwise comparisons of gut microbiota for main factors effect and interaction terms. Analysis is based on Bray Curtis similarity of square root transformed relative abundance data. Permutation method was used on unrestricted permutations of raw data and 999 permutations. A pseudo-F statistic was computed for each permutation and the P (perm) values give the proportion of permuted pseudo-F statistics that are equal to or greater than the original (un-permuted) pseudo-F statistic. A multivariate analogue to the univariate t-statistic was used for the pairwise comparisons. The effect on gut microbiota was tested for factors “treatment” (control and probiotic) and “day” (day 21 and 28). df: Degrees of freedom, SS and MS: Sum and mean of the squares.

**PERMANOVA main test results**						
**Source**	df	SS	MS	Pseudo-F	P(perm)	Unique perms
**Treatment**	1	7269.3	7269.3	8.8805	0.001	998
**Day**	1	6784.7	6784.7	8.2885	0.001	999
**Treatment x Day**	1	2036.1	2036.1	2.4874	0.012	999
**Residual**	44	36017	818.57			
**Total**	47	52107				

**PERMANOVA pairwise comparisons**				
**Term 'Treatment x Day' for pairs of levels of factor 'Treatment'**				
**Within level 'Day 21' of factor 'Day'**				
**Groups**	df	t	P(perm)	Unique perms
**Control vs. Probiotic**	22	2.1573	0.001	996
**Within level 'Day 28' of factor 'Day'**				
**Control vs. Probiotic**	22	2.5883	0.001	999
**Term 'Treatment x Day' for pairs of levels of factor 'Day'**				
**Within level 'Control' of factor 'Treatment'**				
**Day 21 vs 28**	22	1.9031	0.006	996
**Within level 'Probiotic' of factor 'Treatment'**				
**Day 21 vs 28**	22	2.6979	0.001	998

Table S5. Multivariate permutation dispersion (PermDisp) testing for heterogeneity of community structure within each group. Analysis is based on square root relative abundance OTU data. Analysis tested the null hypothesis of no difference between groups' dispersion by computing the dissimilarity of each sample from its group centroid and assessing by permutation analysis whether these dissimilarities differ between the two groups. Significant effects on group dispersion were tested for factor "Treatment" (control & probiotic) on experimental days 21 and 28.

Day 21 of factor "Treatment"	F-statistic	Sample Size	Average Dispersion	
**Control**		12	28.25 (0.73)	
**Probiotic**		12	23.84 (1.32)	
**Deviation from centroid**		t	df	P (perm)
**Control vs. Probiotic**	8.4965	2.915	22	0.011

Day 28 of factor "Treatment"	F-statistic	Sample Size	Average Dispersion	
**Control**		12	27.22 (0.81)	
**Probiotic**		12	24.79 (1.21)	
**Deviation from centroid**		t	df	P (perm)
**Control vs. Probiotic**	2.7827	1.6681	22	0.121

Table S6. Permutational MANOVA table testing for differences in water microbiota between the two treatments on each experimental day. Analysis was based on Bray Curtis similarity of square root transformed relative abundance data. Permutation method was used on unrestricted permutations of raw data. A pseudo-F statistic was computed for each permutation and the P (perm) values give the proportion of permuted pseudo-F statistics that are equal to or greater than the original (un-permuted) pseudo-F statistic. SS and MS: Sum and mean of the squares, df: Degrees of freedom, P(MC): P-values calculated based on 9999 Monte Carlo permutations drawn from the theoretical asymptotic permutation distribution.

PERMANOVA table for factor "Treatment" (Probiotic vs Control)

**Water Day 14**							
**Source**	**df**	**SS**	**MS**	**Pseudo-F**	**P(perm)**	**Unique Perms**	**P(MC)**
**Treatment**	1	1440.9	1440.9	1.3695	**0.19**	10	**0.29**
**Residual**	4	4208.3	1052.1				
**Total**	5	5649.2					
**Water Day 21**							
**Source**	**df**	**SS**	**MS**	**Pseudo-F**	**P(perm)**	**Unique Perms**	**P(MC)**
**Treatment**	1	1197	1197	1.2964	**0.313**	10	**0.311**
**Residual**	4	3693.3	923.33				
**Total**	5	4890.4					
**Water Day 28**							
**Source**	**df**	**SS**	**MS**	**Pseudo-F**	**P(perm)**	**Unique Perms**	**P(MC)**
**Treatment**	1	1440.9	1440.9	1.3695	**0.19**	10	**0.29**
**Residual**	4	4208.3	1052.1				
**Total**	5	5649.2					

Figure S1. Customized cabinet used in probiotic stage. Dimensions LxWxH: 1500x65x70 (mm). (1) HEPA filter (0.3 µm), (2) UV (1 x 30W Ultraviolet 253.7 nm) and daylight (TLD-30W) lamps; (3) Built-in electric sockets, (4) Airtight locks, (5) Gloves, (6) Plexiglas doors (UV-protection), (7) air flow inlet (0.25 µm filter), (8) Custom-built glass chamber (incubation), Ø 12 cm, V: 2L, (9) PVC chamber lid Ø 16 cm, (10) Bottom sieve remover, (11) Heated water inlet, (12 & 13) Outer/inner double wall glass, (14) Heated water outlet and (15) Discharge valve.

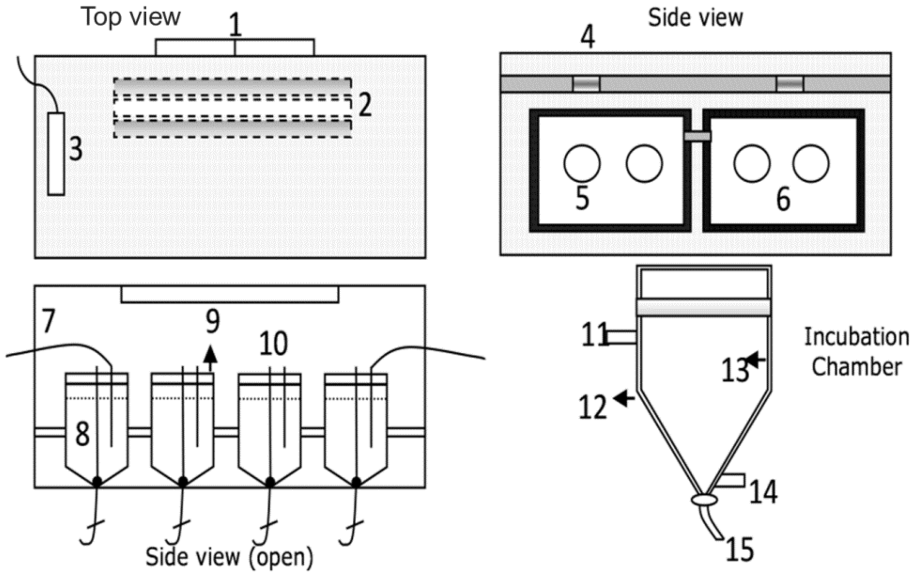

Figure S2. Phylogenetic tree phylogram of *Bacillus* related OTUs and BLAST analysis results for the two most abundant OTUs. Numbers at the branch's end indicate the OTU ID. OTU 814 (blue) is the added *B. subtilis* and 786 (red) is the second most abundant OTU member of the genus *Bacillus* belonging to another species, as indicated in the table. Tree branches give an estimate of a phylogeny where branch lengths are proportional to the amount of inferred evolutionary change.

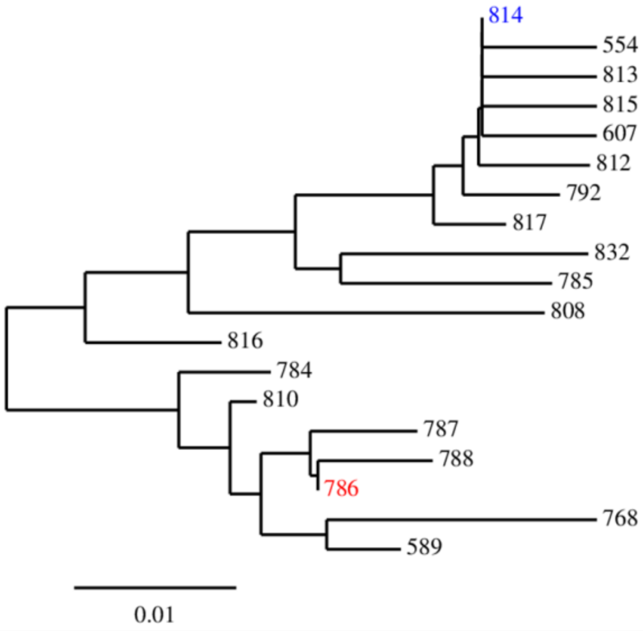

BLASTN 2.2.31+		
RID: N0XKAKB9014		
Database: Nucleotide collection (nt)		
29,980,884 sequences; 93,445,391,531 total letters		
OTU= 814		
Length=141		
Sequences producing significant alignments:	Score E	(Bits) Value
gb|KF318848.1| Bacillus subtilis subsp. subtilis strain I12 1...	134	3.00E-28
gb|KF318844.1| Bacillus subtilis subsp. subtilis strain I8 16...	134	3.00E-28
gb|KF318817.1| Bacillus subtilis subsp. subtilis strain I51 1...	134	3.00E-28
gb|KF318806.1| Bacillus subtilis strain I45 16S ribosomal RNA...	131	3.00E-27
OTU= 786		
Length=141		
Sequences producing significant alignments:	Score E	(Bits) Value
gb|KM205823.1| Bacillus sp. RB05 16S ribosomal RNA gene, part...	136	8.00E-29
gb|KJ606963.1| Uncultured bacterium clone Aer5_12C_T307 16S r...	131	3.00E-27
gb|CP009628.1| Bacillus cereus ATCC 4342, complete genome	131	3.00E-27
gb|KP142408.1| Uncultured bacterium clone PT_1 16S ribosomal ...	131	3.00E-27
gb|JX901106.1| Bacillus cereus strain 8B 16S ribosomal RNA ge...	131	3.00E-27
